# Di­aqua­[5,10,15,20-tetra­kis­(4-chloro­phen­yl)porphyrinato-κ^4^ *N*]iron(III) tri­fluoro­methane­sulfonate–4-hy­droxy-3-meth­oxy­benzaldehyde–water (1/1/2)

**DOI:** 10.1107/S1600536814015335

**Published:** 2014-07-11

**Authors:** Leila Ben Haj Hassen, Khaireddine Ezzayani, Yoann Rousselin, Habib Nasri

**Affiliations:** a Laboratoire de Physico-chimie des Matériaux, Faculté des Sciences de Monastir, Avenue de l’environnement, 5019 Monastir, University of Monastir, Tunisia; b University of Burgundy, ICMUB - UMR 6302, 9 avenue Alain Savary, 21000 Dijon, France

**Keywords:** crystal structure

## Abstract

In the title compound, [Fe(C_44_H_24_Cl_4_N_4_)(H_2_O)_2_](SO_3_CF_3_)·C_8_H_8_O_3_·2H_2_O, the Fe^III^ cation is chelated by the four N atoms of the deprotonated tetra­kis­(4-chloro­tetra­phen­yl)porphyrin (TClPP) and further coordinated by two water mol­ecules in a distorted octa­hedral geometry. In the crystal, the cations, anions, 4-hy­droxy-3-meth­oxy­benzaldehyde and water mol­ecules of crystallization are linked by classical O—H⋯O hydrogen bonds and weak C—H⋯O and C—H⋯Cl hydrogen bonds into a three-dimensional supra­molecular architecture. The crystal packing is further stabilized by weak C—H⋯π inter­actions involving pyrrole and benzene rings. π–π stacking between parallel benzene rings of adjacent 4-hy­droxy-3-meth­oxy­benzaldehyde mol­ecules is also observed, the centroid–centroid distance being 3.8003 (13) Å. The three F atoms of the anion are disordered over two sets of sites, with a refined occupancy ratio 0.527 (12):0.473 (12). The O atom of one water mol­ecule of crystallization is also disordered over two positions in an occupancy ratio of 0.68 (5):0.32 (5).

## Related literature   

For the synthesis, see: Gismelseed *et al.* (1990 ▶). For related structures, see: Gismelseed *et al.* (1990 ▶); Scheidt *et al.* (1979 ▶); Scheidt & Reed (1981 ▶); Scheidt & Finnegan (1989 ▶); Dhifet *et al.* (2009 ▶); Xu *et al.* (2011 ▶); Nasri *et al.* (1990 ▶); Cheng *et al.* (1994 ▶). For a description of the Cambridge Structural Database, see: Allen (2002 ▶).
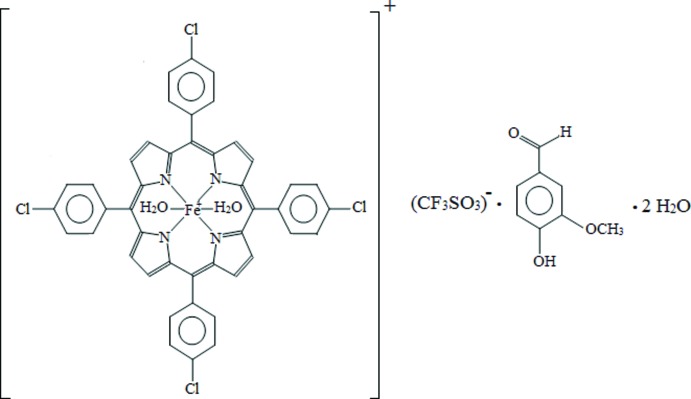


## Experimental   

### 

#### Crystal data  

[Fe(C_44_H_24_Cl_4_N_4_)(H_2_O)_2_](CF_3_O_3_S)·C_8_H_8_O_3_·2H_2_O
*M* *_r_* = 1179.60
Monoclinic, 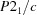

*a* = 10.9998 (4) Å
*b* = 17.8613 (6) Å
*c* = 26.6592 (9) Å
β = 97.9013 (11)°
*V* = 5188.0 (3) Å^3^
*Z* = 4
Mo *K*α radiation
μ = 0.61 mm^−1^
*T* = 115 K
0.2 × 0.2 × 0.1 mm

#### Data collection  

Nonius KappaAPEXII diffractometer
Absorption correction: multi-scan (*SADABS*; Bruker, 2012 ▶) *T*_min_ = 0.885, *T*_max_ = 0.941
95018 measured reflections
11916 independent reflections
8821 reflections with *I* > 2σ(*I*)
*R*_int_ = 0.061

#### Refinement  

*R*[*F* ^2^ > 2σ(*F* ^2^)] = 0.040
*wR*(*F* ^2^) = 0.101
*S* = 1.02
11916 reflections
709 parameters
H-atom parameters constrained
Δρ_max_ = 0.76 e Å^−3^
Δρ_min_ = −0.74 e Å^−3^

### 

Data collection: *APEX2* (Bruker, 2007 ▶); cell refinement: *SAINT* (Bruker, 2007 ▶); data reduction: *SAINT*; program(s) used to solve structure: *OLEX2.solve* (Puschmann *et al.*, 2013 ▶); program(s) used to refine structure: *SHELXTL* (Sheldrick, 2008 ▶); molecular graphics: *ORTEPIII* (Burnett & Johnson, 1996 ▶) and *ORTEP-3 for Windows* (Farrugia, 2012 ▶); software used to prepare material for publication: *OLEX2* (Dolomanov *et al.*, 2009 ▶).

## Supplementary Material

Crystal structure: contains datablock(s) global, I. DOI: 10.1107/S1600536814015335/xu5800sup1.cif

Structure factors: contains datablock(s) I. DOI: 10.1107/S1600536814015335/xu5800Isup2.hkl

CCDC reference: 975656

Additional supporting information:  crystallographic information; 3D view; checkCIF report

## Figures and Tables

**Table 1:** Hydrogen-bond geometry (Å, °) *Cg*2, *Cg*3, *Cg*4, *Cg*10 and *Cg*13 are the centroids of the N2/C40–C43, N3/C30–C33, N4/C19–C22, C2–C7 and C45–C50 rings, respectively.

*D*—H⋯*A*	*D*—H	H⋯*A*	*D*⋯*A*	*D*—H⋯*A*
O1—H1*A*⋯O10*B* ^i^	0.89	1.74	2.626 (8)	171
O1—H1*B*⋯O9	0.89	1.96	2.747 (2)	146
O2—H2*A*⋯O3^ii^	0.88	1.83	2.705 (2)	171
O6—H6*A*⋯O7^iii^	0.87	2.45	3.064 (3)	128
O6—H6*A*⋯O8^iii^	0.87	2.11	2.946 (3)	162
O6—H6*B*⋯O4^iv^	0.87	1.93	2.790 (3)	168
O7—H7*A*⋯O6^v^	0.84	1.77	2.596 (3)	167
O10—H10*A*⋯O7^vi^	0.87	2.07	2.92 (3)	165
O10*B*—H10*C*⋯O5^vii^	0.87	1.92	2.786 (7)	177
O10*B*—H10*D*⋯O7^vi^	0.87	2.02	2.876 (5)	166
C10—H10⋯Cl3^viii^	0.95	2.76	3.659 (2)	159
C14—H14⋯O5^ix^	0.95	2.38	3.297 (3)	162
C31—H31⋯Cl2^i^	0.95	2.82	3.739 (2)	163
C4*EA*—H4*EA*⋯*Cg*4^x^	0.95	2.66	3.5054	149
C17—H17⋯*Cg*2^ii^	0.95	2.76	3.5715	144
C20—H20⋯*Cg*13^ii^	0.95	2.82	3.5054	130
C28—H28⋯*Cg*3^xi^	0.95	2.79	3.6574	152
C37—H37⋯*Cg*10^xii^	0.95	2.76	3.6107	149

Symmetry codes: (i) 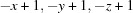; (ii) 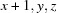; (iii) 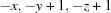; (iv) 
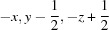
; (v) 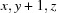; (vi) 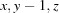; (vii) 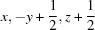; (viii) 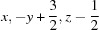; (ix) 
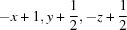
; (x) 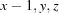; (xi) 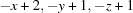; (xii) 
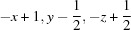
.

**Fig. 1.**
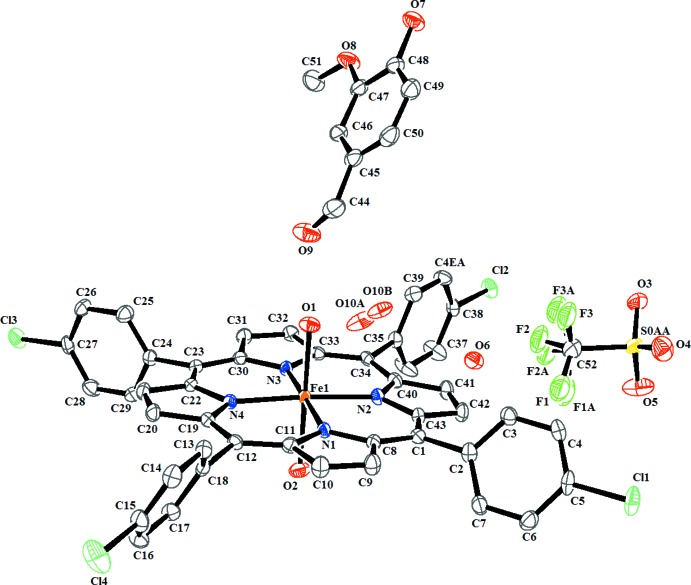
An ORTEP view of the molecular structure of the title molecule with the atom-numbering. Displacement ellipsoids are drawn at 60%. The H atoms have been omitted for clarity.

**Fig. 2.**
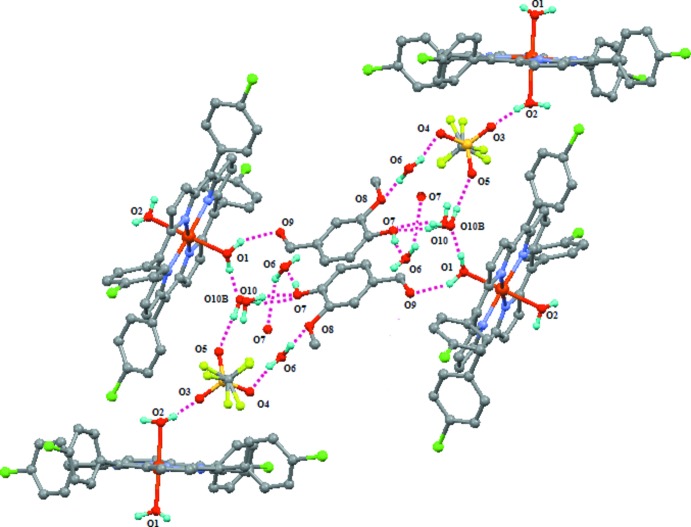
A general view of the crystal packing for the title compound with hydrogen bonds drawn as dashed lines. Only the major position O10 of the oxygen atom of one disordered water molecule is shown.

## supplementary crystallographic information

### S0.1. Refinement

The positions of H atoms of the two aqua ligands were found in difference maps and then refine with U_iso_(H) = 1.5U_eq_(O). The H atoms of the two water molecules were placed in calculated positions with a distances restraint of O–H = 0.87 Å, with U_iso_(H)= 1.5U_eq_(O). All other H atoms were placed in geometrically idealized positions with C—H = 0.95-0.98 Å and constrained to ride on their parent atoms, U_iso_(H) = 1.2U_eq_(C).

The three fluorine atoms of the triflate counterion are disordered over two orientations [F1—F2—F3 / F1A—F2A—F3A] with refined occupancy coefficients converged to 0.473 (12) and 0.527 (12). EADP of SHELXL97 (Sheldrick, 2008) commands were used to model the disorder for fluorine atoms in triflate counterion. The oxygen atom of one water molecule is disordered over two positions [O10/O10B] in a 0.32 (5):0.68 (5) ratio.

### S0.2. Synthesis and crystallization

To a solution of [Fe^III^(TClPP)(SO_3_CF_3_)] (Gismelseed *et al.*, 1990) (15 mg, 0.0156 mmol) in chloro­form (15 mL) was added an excess of 4-hy­droxy-3-méthoxybenzaldéhyde (vanilline) (100 mg, 0.657 mmol). The reaction mixture was stirred at room temperature and at the end of the reaction, the color of the solution changes from brown red to blood red. The resulting material was crystallized by diffusion of hexanes through the chloro­form solution which yielded [Fe^III^(C_44_H_28_Cl_4_N_4_)(H_2_O)_2_](SO_3_CF_3_).(C_8_H_8_O_3_).2(H_2_O). The X-ray analysis was recorded in the "Pôle de Chimie Moléculaire", the technological platform for chemical analysis and molecular synthesis (http://www.wpcm.fr) which relies on the Institute of the Molecular Chemistry of University of Burgundy and Welience "TM", a Burgundy University private subsidiary.

### S1. Comment

An extensive number of molecular structures of iron(III) porphyrin complexes is reported in the literature. Nevertheless in the Cambridge Structural Database (CSD, Version 5.35; Allen, 2002) there are only thirteen reported structures of aqua-iron(III) *meso-*porphyrins or *β-pyrrolic-*porphrin complexes. One other di­aqua structure of the coordination compound [Fe^III^(TPP)(H_2_O)_2_](ClO_4_).2THF was published in 1979 (Scheidt *et al.*, 1979). Among these iron(III)-aqua porphyrins structures, there are four mono-aqua, four di­aqua and six mixed-ligands "aqua-L" molecular structures (L is a monodentate ligand).

We reports herein the crystal structure of the di­aqua­(5, 10, 15, 20-tetra­(para-chloro­phenyl)­porphyrinato-κ^4^*N*)iron(III) tri­fluoro­methane­sulfonate 4-hy­droxy-3-meth­oxy­benzaldehyde dihydrate with formula [Fe^III^(TClPP)(H_2_O)_2_](SO_3_CF_3_).(C_8_H_8_O_3_).2(H_2_O) (where TClPP is the dianion of the 5, 10, 15, 20-tetra­(para-chloro­phenyl)­porphyrin).

In this complex, the iron is coordinated to the four N atoms of the porphyrin ring and the oxygen atoms of the two trans aqua axial ligands (Fig. 1).

It has been noticed for iron(III) porphyrins that there is a relationship between the spin-state for the iron(III) and the value of the average equatorial iron-pyrrole N atoms distance (Fe—Np) (Scheidt & Reed, 1981; Cheng *et al.*, 1994). Generally, the spin-state of the Fe(III) porphyrins depends on the value of the Fe—Np bond length. Thus, for the high-spin state (S = 5/2) species, the Fe—Np distance values are large i.e, for the [Fe^III^(TPP)Cl] complex, Fe—Np = 2.070 (9) Å (Scheidt & Finnegan, 1989) and 2.125 (2) Å for the [Fe^III^(TpivPP)(η^2^-O_2_CO)]^-^ species (Dhifet *et al.*, 2009). For low-spin state (S = 1/2), the Fe—Np bond length is smaller than those of high-spin Fe(III) porphyrins, i.e, for the [Fe^III^(TpivPP)(NO_2_)_2_]^-^ species, the Fe—Np distance is 1.992 (1) Å (Nasri *et al.*, 1990). The inter­medied-spin (S = 3/2) Fe(III) porphyrin complexes present the smallest Fe—Np distances, i.e, the [Fe^III^(TPP)(3-Clpy)]ClO_4_ exhibits an Fe—Np bond length of 1.979 (6) Å. Admixed inter­mediate-spin [S = (5/2,3/2)] Fe(III) porphyrins display Fe—Np value around 2.000 Å as in the case of the [Fe^III^(TPP)(ClO_4_)] complex [Fe—Np = 2.022 (8) Å] (Gismelseed *et al.*, 1990, Refcode SICFAL).

The Fe—Np distance value of our derivative [Fe^III^(TClPP)(H_2_O)_2_]^+^ which is 2.042 (2) Å is an indication that this species is high-spin (S = 5/2).

It is noteworthy that Fe(III)-mono­aqua porphyrins are inter­mediate-spin (S = 3/2) with an Fe—Np distances around 1.978 Å while Fe(III)-di­aqua metalloporphyrins are high-spin (S = 5/2) with an Fe—Np distance around 2.045 Å (Cheng *et al.*, 1994) . Thus, for [Fe^III^(TPP)(H_2_O)]^+^ (Xu *et al.*, 2011), the Fe—Np distance is 1.982 (3) Å and the [Fe^III^(TPP)(H_2_O)_2_]^+^ complex exhibits a Fe—Np distance of 2.045 (8) Å (Scheidt *et al.*, 1979). For Fe(III) mixed-ligands porphyrins type [Fe^III^(Porph)(H_2_O)(L)]^+^ (Porph = porphyrinato) the spin state depends on the nature of the axial L ligand. For example, the Fe—Np distance is 2.022 (8) Å for [Fe^III^(TpivPP)(SO_3_CF_3_)(H_2_O)] leading to an admixed inter­mediate-spin derivative [S = (5/2,3/2)] (Gismelseed *et al.*, 1990).

For our iron(III) derivative, the axial Fe—O(H_2_O) bond lengths are 2.051 (2) Å and 2.157 (2) Å while for the [Fe^III^(TPP)(H_2_O)_2_]^+^ related species (Scheidt *et al.*, 1979), this distance is 2.095 (2) Å. These bond lengths values are comparable to those of several iron(III)-aqua porphyrin complexes [1.961 (3) Å - 2.134 (6) Å] (CSD refcodes ECADET; Xu *et al.*, 2011 and SICFAL; Gismelseed *et al.*, 1990) (CDS, version 5.35, Allen, 2002).

The porphyrin ring is far from being planar, with deviations of atoms from the leasts squares plane ranging from -0.162 (2) Å to 0.110 (2) Å.

In the crystal structure (Fig. 2), The oxygen O3 of the triflate counterion (SO_3_CF_3_)^-^ is linked by strong hydrogen bonds to the oxygen atom O2 of one water molecule coordinated to the iron(III) and the two non-coordinated water molecules (O10 and O6 oxygens). The later molecules are also hydrogen bonded to the vanilline molecule through oxygens O7 and O8. On the other hand, this vanilline molecule is also bonded by H bond to the oxygen O1 of one water molecule coordinated to the iron(III).

The crystal is further consolidated by C—H···.π inter­molecular inter­actions involving Cg pyrrole and phenyl centroids rings (Table 2).

### Crystal data

**Table:**

[Fe(C_44_H_24_Cl_4_N_4_)(H_2_O)_2_](CF_3_O_3_S)·C_8_H_8_O_3_·2H_2_O	*F*(000) = 2412
*M**_r_* = 1179.60	*D*_x_ = 1.510 Mg m^−^^3^
Monoclinic, *P*2_1_/*c*	Mo *K*α radiation, λ = 0.71073 Å
*a* = 10.9998 (4) Å	Cell parameters from 9872 reflections
*b* = 17.8613 (6) Å	θ = 2.5–27.4°
*c* = 26.6592 (9) Å	µ = 0.61 mm^−^^1^
β = 97.9013 (11)°	*T* = 115 K
*V* = 5188.0 (3) Å^3^	Prism, dark violet
*Z* = 4	0.2 × 0.2 × 0.1 mm

### Data collection

**Table:**

Nonius KappaAPEXII diffractometer	11916 independent reflections
Radiation source: X-ray tube, Siemens KFF Mo 2K-180	8821 reflections with *I* > 2σ(*I*)
Graphite monochromator	*R*_int_ = 0.061
φ and ω scans	θ_max_ = 27.5°, θ_min_ = 2.4°
Absorption correction: multi-scan (*SADABS*; Bruker, 2012)	*h* = −14→14
*T*_min_ = 0.885, *T*_max_ = 0.941	*k* = −23→23
95018 measured reflections	*l* = −34→34

### Refinement

**Table:**

Refinement on *F*^2^	6 constraints
Least-squares matrix: full	Hydrogen site location: inferred from neighbouring sites
*R*[*F*^2^ > 2σ(*F*^2^)] = 0.040	H-atom parameters constrained
*wR*(*F*^2^) = 0.101	*w* = 1/[σ^2^(*F*_o_^2^) + (0.0399*P*)^2^ + 6.1729*P*] where *P* = (*F*_o_^2^ + 2*F*_c_^2^)/3
*S* = 1.02	(Δ/σ)_max_ = 0.002
11916 reflections	Δρ_max_ = 0.76 e Å^−^^3^
709 parameters	Δρ_min_ = −0.74 e Å^−^^3^
0 restraints	

### Special details

**Table:**

Geometry. All e.s.d.'s (except the e.s.d. in the dihedral angle between two l.s. planes) are estimated using the full covariance matrix. The cell e.s.d.'s are taken into account individually in the estimation of e.s.d.'s in distances, angles and torsion angles; correlations between e.s.d.'s in cell parameters are only used when they are defined by crystal symmetry. An approximate (isotropic) treatment of cell e.s.d.'s is used for estimating e.s.d.'s involving l.s. planes.

### Fractional atomic coordinates and isotropic or equivalent isotropic displacement parameters (Å^2^)

**Table:**

	*x*	*y*	*z*	*U*_iso_*/*U*_eq_	Occ. (<1)
C1	0.7091 (2)	0.75273 (13)	0.22709 (8)	0.0148 (4)	
C2	0.6504 (2)	0.76670 (13)	0.17385 (8)	0.0159 (5)	
C3	0.5374 (2)	0.80365 (13)	0.16516 (9)	0.0167 (5)	
H3	0.4995	0.8203	0.1931	0.020*	
C4	0.4800 (2)	0.81620 (13)	0.11625 (9)	0.0189 (5)	
H4	0.4027	0.8408	0.1106	0.023*	
C4EA	0.2576 (2)	0.59674 (13)	0.39721 (9)	0.0193 (5)	
H4EA	0.1928	0.6270	0.4061	0.023*	
C5	0.5364 (2)	0.79256 (14)	0.07589 (9)	0.0193 (5)	
C6	0.6483 (2)	0.75627 (14)	0.08291 (9)	0.0211 (5)	
H6	0.6860	0.7405	0.0547	0.025*	
C7	0.7051 (2)	0.74313 (14)	0.13225 (9)	0.0197 (5)	
H7	0.7818	0.7179	0.1376	0.024*	
C8	0.8235 (2)	0.78571 (12)	0.24315 (8)	0.0135 (4)	
C9	0.8903 (2)	0.83203 (14)	0.21212 (9)	0.0192 (5)	
H9	0.8630	0.8477	0.1784	0.023*	
C10	0.9989 (2)	0.84900 (14)	0.23989 (8)	0.0194 (5)	
H10	1.0626	0.8781	0.2290	0.023*	
C11	1.0008 (2)	0.81518 (12)	0.28879 (8)	0.0140 (4)	
C12	1.0996 (2)	0.81831 (12)	0.32806 (8)	0.0133 (4)	
C13	1.2167 (2)	0.85384 (13)	0.31672 (8)	0.0143 (4)	
C14	1.2242 (2)	0.92948 (13)	0.30468 (8)	0.0165 (5)	
H14	1.1539	0.9605	0.3042	0.020*	
C15	1.3339 (2)	0.96011 (14)	0.29332 (9)	0.0193 (5)	
H15	1.3381	1.0115	0.2846	0.023*	
C16	1.4362 (2)	0.91480 (14)	0.29486 (9)	0.0205 (5)	
C17	1.4318 (2)	0.83969 (14)	0.30698 (9)	0.0201 (5)	
H17	1.5030	0.8093	0.3082	0.024*	
C18	1.3217 (2)	0.80948 (13)	0.31731 (8)	0.0161 (5)	
H18	1.3175	0.7577	0.3249	0.019*	
C19	1.0996 (2)	0.78766 (12)	0.37654 (8)	0.0131 (4)	
C20	1.1960 (2)	0.79639 (13)	0.41813 (8)	0.0161 (5)	
H20	1.2700	0.8237	0.4178	0.019*	
C21	1.1622 (2)	0.75870 (13)	0.45793 (8)	0.0159 (5)	
H21	1.2083	0.7545	0.4907	0.019*	
C22	1.0437 (2)	0.72617 (12)	0.44171 (8)	0.0131 (4)	
C23	0.9787 (2)	0.68094 (12)	0.47167 (8)	0.0135 (4)	
C24	1.0435 (2)	0.65774 (13)	0.52273 (8)	0.0140 (4)	
C25	1.0415 (2)	0.70133 (13)	0.56561 (9)	0.0196 (5)	
H25	0.9967	0.7470	0.5632	0.024*	
C26	1.1043 (2)	0.67914 (14)	0.61238 (8)	0.0197 (5)	
H26	1.1035	0.7095	0.6416	0.024*	
C27	1.1674 (2)	0.61222 (14)	0.61515 (8)	0.0173 (5)	
C28	1.1707 (2)	0.56756 (14)	0.57324 (9)	0.0216 (5)	
H28	1.2149	0.5217	0.5760	0.026*	
C29	1.1086 (2)	0.59058 (14)	0.52690 (9)	0.0187 (5)	
H29	1.1105	0.5602	0.4978	0.022*	
C30	0.8601 (2)	0.65259 (12)	0.45727 (8)	0.0133 (4)	
C31	0.7898 (2)	0.61143 (13)	0.48957 (8)	0.0158 (5)	
H31	0.8160	0.5974	0.5237	0.019*	
C32	0.6794 (2)	0.59620 (13)	0.46239 (8)	0.0154 (5)	
H32	0.6135	0.5701	0.4740	0.019*	
C33	0.6804 (2)	0.62697 (12)	0.41259 (8)	0.0130 (4)	
C34	0.5852 (2)	0.62091 (12)	0.37230 (8)	0.0134 (4)	
C35	0.4675 (2)	0.58381 (13)	0.38095 (8)	0.0147 (5)	
C36	0.4471 (2)	0.50808 (14)	0.37200 (11)	0.0281 (6)	
H36	0.5120	0.4773	0.3637	0.034*	
C37	0.3326 (2)	0.47665 (14)	0.37508 (11)	0.0280 (6)	
H37	0.3190	0.4247	0.3690	0.034*	
C38	0.2394 (2)	0.52161 (13)	0.38702 (8)	0.0156 (5)	
C39	0.3724 (2)	0.62737 (13)	0.39432 (9)	0.0174 (5)	
H39	0.3862	0.6790	0.4016	0.021*	
C40	0.5879 (2)	0.64867 (12)	0.32326 (8)	0.0143 (4)	
C41	0.4945 (2)	0.63557 (13)	0.28105 (9)	0.0177 (5)	
H41	0.4215	0.6073	0.2816	0.021*	
C42	0.5291 (2)	0.67066 (13)	0.24049 (9)	0.0178 (5)	
H42	0.4858	0.6710	0.2071	0.021*	
C43	0.6443 (2)	0.70764 (12)	0.25718 (8)	0.0140 (4)	
N1	0.89170 (16)	0.77736 (10)	0.29038 (7)	0.0125 (4)	
N2	0.67932 (17)	0.69230 (10)	0.30781 (7)	0.0133 (4)	
N3	0.79152 (16)	0.66139 (10)	0.41031 (7)	0.0122 (4)	
N4	1.00622 (16)	0.74539 (10)	0.39191 (7)	0.0128 (4)	
O1	0.76299 (15)	0.81594 (9)	0.37720 (6)	0.0187 (3)	
H1A	0.8187	0.8522	0.3839	0.028*	
H1B	0.7319	0.8059	0.4057	0.028*	
O2	0.91879 (16)	0.62112 (9)	0.32428 (6)	0.0216 (4)	
H2A	0.9100	0.6203	0.2909	0.032*	
H2B	0.8663	0.5905	0.3356	0.032*	
Cl1	0.46219 (6)	0.80909 (4)	0.01479 (2)	0.02837 (15)	
Cl2	0.09344 (5)	0.48365 (3)	0.38831 (2)	0.02059 (13)	
Cl3	1.24603 (6)	0.58219 (4)	0.67323 (2)	0.02717 (15)	
Cl4	1.57458 (6)	0.95266 (4)	0.28187 (3)	0.03687 (18)	
Fe1	0.84010 (3)	0.72182 (2)	0.35080 (2)	0.01104 (8)	
C44	0.5613 (2)	0.89392 (16)	0.43434 (10)	0.0278 (6)	
H44	0.5868	0.9163	0.4052	0.033*	
C45	0.4549 (2)	0.92685 (14)	0.45329 (9)	0.0223 (5)	
C46	0.4162 (2)	0.90025 (14)	0.49835 (9)	0.0213 (5)	
H46	0.4613	0.8623	0.5178	0.026*	
C47	0.3121 (2)	0.93010 (14)	0.51372 (9)	0.0204 (5)	
C48	0.2449 (2)	0.98641 (14)	0.48501 (9)	0.0211 (5)	
C49	0.2843 (2)	1.01260 (15)	0.44081 (9)	0.0249 (5)	
H49	0.2397	1.0507	0.4214	0.030*	
C50	0.3895 (2)	0.98248 (15)	0.42550 (9)	0.0247 (6)	
H50	0.4169	1.0004	0.3954	0.030*	
C51	0.3268 (3)	0.85316 (16)	0.58774 (10)	0.0322 (6)	
H51A	0.3313	0.8073	0.5679	0.048*	
H51B	0.2826	0.8429	0.6165	0.048*	
H51C	0.4099	0.8705	0.6002	0.048*	
O7	0.14350 (17)	1.01146 (11)	0.50295 (6)	0.0269 (4)	
H7A	0.1067	1.0422	0.4824	0.040*	
O8	0.26317 (17)	0.90970 (11)	0.55642 (7)	0.0281 (4)	
O9	0.62056 (18)	0.84012 (11)	0.45260 (8)	0.0351 (5)	
C52	0.1484 (3)	0.61624 (19)	0.22889 (11)	0.0362 (7)	
O3	−0.08765 (17)	0.60737 (11)	0.22290 (7)	0.0312 (4)	
O4	−0.00031 (18)	0.65653 (11)	0.15053 (7)	0.0342 (5)	
O5	0.0170 (2)	0.52536 (11)	0.17085 (8)	0.0390 (5)	
S0AA	0.00338 (6)	0.60012 (3)	0.18920 (2)	0.01966 (13)	
O6	0.00468 (17)	0.10763 (11)	0.44911 (7)	0.0289 (4)	
H6A	−0.0702	0.0969	0.4537	0.043*	
H6B	0.0071	0.1162	0.4171	0.043*	
O10	0.149 (5)	0.0536 (15)	0.6090 (10)	0.044 (8)	0.32 (5)
H10A	0.1607	0.0449	0.5779	0.066*	0.32 (5)
H10B	0.1076	0.0166	0.6198	0.066*	0.32 (5)
O10B	0.0901 (17)	0.0701 (5)	0.5980 (4)	0.034 (2)	0.68 (5)
H10C	0.0653	0.0397	0.6199	0.050*	0.68 (5)
H10D	0.1034	0.0452	0.5713	0.050*	0.68 (5)
F1_1	0.2374 (6)	0.6047 (3)	0.19998 (16)	0.0441 (12)	0.473 (12)
F2_1	0.1574 (4)	0.6952 (3)	0.2380 (2)	0.0441 (12)	0.473 (12)
F3_1	0.1719 (4)	0.5850 (4)	0.27147 (15)	0.0441 (12)	0.473 (12)
F1A_2	0.2464 (6)	0.6124 (3)	0.20575 (19)	0.0448 (11)	0.527 (12)
F2A_2	0.1492 (4)	0.6758 (3)	0.2566 (2)	0.0448 (11)	0.527 (12)
F3A_2	0.1597 (4)	0.5563 (4)	0.26316 (17)	0.0448 (11)	0.527 (12)

### Atomic displacement parameters (Å^2^)

**Table:**

	*U*^11^	*U*^22^	*U*^33^	*U*^12^	*U*^13^	*U*^23^
C1	0.0134 (11)	0.0175 (11)	0.0136 (10)	0.0021 (9)	0.0019 (8)	0.0003 (9)
C2	0.0138 (11)	0.0182 (12)	0.0155 (11)	−0.0042 (9)	0.0007 (9)	0.0045 (9)
C3	0.0131 (11)	0.0188 (12)	0.0183 (11)	−0.0022 (9)	0.0022 (9)	0.0005 (9)
C4	0.0121 (11)	0.0208 (12)	0.0222 (12)	−0.0009 (9)	−0.0030 (9)	0.0043 (10)
C4EA	0.0146 (12)	0.0217 (12)	0.0228 (12)	0.0003 (10)	0.0073 (9)	−0.0018 (10)
C5	0.0177 (12)	0.0238 (13)	0.0141 (11)	−0.0067 (10)	−0.0054 (9)	0.0065 (9)
C6	0.0203 (12)	0.0287 (13)	0.0146 (11)	−0.0031 (10)	0.0030 (9)	−0.0013 (10)
C7	0.0137 (11)	0.0267 (13)	0.0180 (11)	0.0014 (10)	−0.0005 (9)	0.0015 (10)
C8	0.0119 (11)	0.0168 (11)	0.0115 (10)	0.0007 (9)	0.0015 (8)	0.0004 (8)
C9	0.0169 (12)	0.0267 (13)	0.0135 (11)	−0.0029 (10)	0.0007 (9)	0.0056 (9)
C10	0.0168 (12)	0.0259 (13)	0.0157 (11)	−0.0046 (10)	0.0030 (9)	0.0044 (10)
C11	0.0133 (11)	0.0155 (11)	0.0137 (10)	−0.0010 (9)	0.0037 (8)	0.0004 (9)
C12	0.0099 (10)	0.0150 (11)	0.0154 (11)	−0.0014 (9)	0.0032 (8)	−0.0013 (9)
C13	0.0131 (11)	0.0195 (12)	0.0100 (10)	−0.0039 (9)	0.0006 (8)	−0.0024 (9)
C14	0.0148 (11)	0.0194 (12)	0.0147 (11)	0.0002 (9)	−0.0001 (9)	−0.0003 (9)
C15	0.0213 (13)	0.0178 (12)	0.0182 (11)	−0.0055 (10)	0.0005 (9)	0.0023 (9)
C16	0.0142 (12)	0.0285 (13)	0.0190 (12)	−0.0079 (10)	0.0034 (9)	0.0021 (10)
C17	0.0145 (12)	0.0253 (13)	0.0212 (12)	0.0004 (10)	0.0050 (9)	−0.0001 (10)
C18	0.0160 (11)	0.0158 (11)	0.0169 (11)	−0.0018 (9)	0.0042 (9)	0.0003 (9)
C19	0.0105 (10)	0.0152 (11)	0.0135 (10)	0.0005 (9)	0.0020 (8)	−0.0008 (8)
C20	0.0123 (11)	0.0196 (12)	0.0159 (11)	−0.0036 (9)	0.0004 (9)	−0.0022 (9)
C21	0.0137 (11)	0.0210 (12)	0.0123 (10)	−0.0015 (9)	−0.0008 (8)	−0.0018 (9)
C22	0.0120 (10)	0.0171 (11)	0.0101 (10)	0.0019 (9)	0.0009 (8)	−0.0019 (8)
C23	0.0125 (11)	0.0159 (11)	0.0119 (10)	0.0018 (9)	0.0013 (8)	−0.0019 (8)
C24	0.0112 (11)	0.0185 (11)	0.0126 (10)	−0.0033 (9)	0.0031 (8)	0.0020 (9)
C25	0.0231 (13)	0.0184 (12)	0.0168 (11)	0.0023 (10)	0.0012 (10)	−0.0002 (9)
C26	0.0217 (12)	0.0258 (13)	0.0110 (10)	−0.0002 (10)	0.0008 (9)	−0.0025 (9)
C27	0.0100 (11)	0.0297 (13)	0.0118 (10)	0.0001 (10)	0.0003 (8)	0.0058 (9)
C28	0.0197 (13)	0.0261 (13)	0.0191 (12)	0.0072 (10)	0.0036 (10)	0.0037 (10)
C29	0.0185 (12)	0.0247 (13)	0.0132 (11)	0.0031 (10)	0.0027 (9)	−0.0023 (9)
C30	0.0140 (11)	0.0143 (11)	0.0118 (10)	0.0021 (9)	0.0022 (8)	−0.0002 (8)
C31	0.0154 (11)	0.0182 (12)	0.0140 (11)	−0.0010 (9)	0.0027 (9)	0.0015 (9)
C32	0.0145 (11)	0.0165 (11)	0.0160 (11)	−0.0021 (9)	0.0048 (9)	0.0017 (9)
C33	0.0122 (11)	0.0124 (10)	0.0148 (11)	−0.0006 (8)	0.0037 (8)	−0.0006 (8)
C34	0.0093 (10)	0.0127 (11)	0.0186 (11)	−0.0009 (8)	0.0029 (8)	0.0003 (9)
C35	0.0126 (11)	0.0176 (11)	0.0141 (10)	−0.0025 (9)	0.0020 (8)	0.0030 (9)
C36	0.0150 (12)	0.0176 (12)	0.0531 (17)	0.0019 (10)	0.0101 (12)	−0.0008 (12)
C37	0.0181 (13)	0.0131 (12)	0.0540 (18)	−0.0043 (10)	0.0091 (12)	−0.0024 (11)
C38	0.0106 (11)	0.0208 (12)	0.0153 (11)	−0.0047 (9)	0.0009 (8)	0.0044 (9)
C39	0.0177 (12)	0.0160 (11)	0.0191 (11)	−0.0040 (9)	0.0052 (9)	−0.0021 (9)
C40	0.0110 (11)	0.0149 (11)	0.0172 (11)	−0.0004 (9)	0.0022 (9)	0.0002 (9)
C41	0.0121 (11)	0.0206 (12)	0.0193 (11)	−0.0041 (9)	−0.0012 (9)	0.0005 (9)
C42	0.0133 (11)	0.0223 (12)	0.0166 (11)	−0.0037 (9)	−0.0022 (9)	−0.0012 (9)
C43	0.0114 (11)	0.0162 (11)	0.0138 (10)	0.0003 (9)	−0.0011 (8)	0.0000 (8)
N1	0.0091 (9)	0.0166 (9)	0.0117 (9)	−0.0006 (7)	0.0010 (7)	0.0006 (7)
N2	0.0109 (9)	0.0154 (9)	0.0135 (9)	−0.0006 (7)	0.0008 (7)	0.0018 (7)
N3	0.0099 (9)	0.0153 (9)	0.0112 (9)	−0.0013 (7)	0.0012 (7)	0.0003 (7)
N4	0.0106 (9)	0.0170 (9)	0.0110 (9)	−0.0008 (7)	0.0021 (7)	−0.0004 (7)
O1	0.0178 (9)	0.0177 (8)	0.0225 (9)	−0.0001 (7)	0.0089 (7)	−0.0012 (7)
O2	0.0272 (10)	0.0221 (9)	0.0164 (8)	0.0054 (7)	0.0065 (7)	−0.0012 (7)
Cl1	0.0245 (3)	0.0417 (4)	0.0164 (3)	−0.0080 (3)	−0.0063 (2)	0.0101 (3)
Cl2	0.0119 (3)	0.0247 (3)	0.0254 (3)	−0.0047 (2)	0.0032 (2)	0.0052 (2)
Cl3	0.0195 (3)	0.0486 (4)	0.0133 (3)	0.0091 (3)	0.0017 (2)	0.0083 (3)
Cl4	0.0188 (3)	0.0420 (4)	0.0512 (4)	−0.0106 (3)	0.0098 (3)	0.0146 (3)
Fe1	0.00874 (15)	0.01416 (16)	0.01025 (14)	−0.00075 (12)	0.00145 (11)	0.00049 (12)
C44	0.0209 (13)	0.0363 (16)	0.0282 (14)	−0.0101 (12)	0.0108 (11)	−0.0109 (12)
C45	0.0167 (12)	0.0276 (13)	0.0228 (12)	−0.0064 (10)	0.0038 (10)	−0.0108 (10)
C46	0.0180 (12)	0.0230 (13)	0.0227 (12)	−0.0004 (10)	0.0024 (10)	−0.0071 (10)
C47	0.0202 (12)	0.0257 (13)	0.0156 (11)	−0.0019 (10)	0.0037 (9)	−0.0060 (10)
C48	0.0180 (12)	0.0269 (13)	0.0189 (12)	0.0003 (10)	0.0040 (9)	−0.0058 (10)
C49	0.0241 (13)	0.0304 (14)	0.0201 (12)	−0.0008 (11)	0.0026 (10)	−0.0015 (11)
C50	0.0228 (13)	0.0347 (15)	0.0176 (12)	−0.0087 (11)	0.0062 (10)	−0.0058 (11)
C51	0.0333 (16)	0.0409 (17)	0.0228 (13)	0.0130 (13)	0.0055 (12)	0.0078 (12)
O7	0.0252 (10)	0.0352 (11)	0.0219 (9)	0.0115 (8)	0.0086 (8)	0.0060 (8)
O8	0.0278 (10)	0.0369 (11)	0.0217 (9)	0.0136 (8)	0.0109 (8)	0.0063 (8)
O9	0.0289 (11)	0.0360 (11)	0.0441 (12)	0.0035 (9)	0.0185 (9)	−0.0037 (9)
C52	0.0267 (15)	0.057 (2)	0.0242 (14)	0.0059 (14)	0.0021 (12)	−0.0089 (14)
O3	0.0255 (10)	0.0480 (12)	0.0209 (9)	0.0048 (9)	0.0065 (8)	−0.0075 (8)
O4	0.0347 (11)	0.0356 (11)	0.0319 (10)	0.0032 (9)	0.0028 (9)	0.0093 (9)
O5	0.0449 (13)	0.0284 (11)	0.0494 (13)	−0.0089 (9)	0.0271 (10)	−0.0135 (9)
S0AA	0.0197 (3)	0.0235 (3)	0.0162 (3)	−0.0002 (2)	0.0040 (2)	−0.0025 (2)
O6	0.0299 (11)	0.0314 (10)	0.0266 (10)	0.0053 (9)	0.0079 (8)	0.0060 (8)
O10	0.074 (18)	0.027 (7)	0.038 (6)	−0.026 (9)	0.032 (9)	−0.011 (5)
O10B	0.052 (6)	0.024 (2)	0.030 (3)	−0.013 (3)	0.021 (3)	−0.0083 (18)
F1_1	0.0317 (16)	0.072 (2)	0.0259 (16)	−0.0074 (13)	−0.0058 (10)	−0.0084 (12)
F2_1	0.0317 (16)	0.072 (2)	0.0259 (16)	−0.0074 (13)	−0.0058 (10)	−0.0084 (12)
F3_1	0.0317 (16)	0.072 (2)	0.0259 (16)	−0.0074 (13)	−0.0058 (10)	−0.0084 (12)
F1A_2	0.0330 (14)	0.071 (2)	0.0279 (15)	−0.0038 (12)	−0.0032 (10)	−0.0091 (11)
F2A_2	0.0330 (14)	0.071 (2)	0.0279 (15)	−0.0038 (12)	−0.0032 (10)	−0.0091 (11)
F3A_2	0.0330 (14)	0.071 (2)	0.0279 (15)	−0.0038 (12)	−0.0032 (10)	−0.0091 (11)

### Geometric parameters (Å, º)

**Table:**

C1—C2	1.497 (3)	C33—C34	1.398 (3)
C1—C8	1.402 (3)	C33—N3	1.377 (3)
C1—C43	1.399 (3)	C34—C35	1.500 (3)
C2—C3	1.399 (3)	C34—C40	1.402 (3)
C2—C7	1.397 (3)	C35—C36	1.386 (3)
C3—H3	0.9500	C35—C39	1.389 (3)
C3—C4	1.386 (3)	C36—H36	0.9500
C4—H4	0.9500	C36—C37	1.392 (3)
C4—C5	1.380 (3)	C37—H37	0.9500
C4EA—H4EA	0.9500	C37—C38	1.373 (3)
C4EA—C38	1.378 (3)	C38—Cl2	1.747 (2)
C4EA—C39	1.388 (3)	C39—H39	0.9500
C5—C6	1.381 (3)	C40—C41	1.435 (3)
C5—Cl1	1.744 (2)	C40—N2	1.379 (3)
C6—H6	0.9500	C41—H41	0.9500
C6—C7	1.396 (3)	C41—C42	1.349 (3)
C7—H7	0.9500	C42—H42	0.9500
C8—C9	1.441 (3)	C42—C43	1.443 (3)
C8—N1	1.382 (3)	C43—N2	1.379 (3)
C9—H9	0.9500	N1—Fe1	2.0377 (18)
C9—C10	1.351 (3)	N2—Fe1	2.0402 (18)
C10—H10	0.9500	N3—Fe1	2.0497 (18)
C10—C11	1.434 (3)	N4—Fe1	2.0412 (18)
C11—C12	1.403 (3)	O1—H1A	0.8932
C11—N1	1.383 (3)	O1—H1B	0.8931
C12—C13	1.504 (3)	O1—Fe1	2.0506 (16)
C12—C19	1.404 (3)	O2—H2A	0.8809
C13—C14	1.394 (3)	O2—H2B	0.8779
C13—C18	1.398 (3)	O2—Fe1	2.1570 (16)
C14—H14	0.9500	C44—H44	0.9500
C14—C15	1.395 (3)	C44—C45	1.460 (3)
C15—H15	0.9500	C44—O9	1.224 (3)
C15—C16	1.383 (3)	C45—C46	1.411 (3)
C16—C17	1.382 (3)	C45—C50	1.381 (4)
C16—Cl4	1.743 (2)	C46—H46	0.9500
C17—H17	0.9500	C46—C47	1.376 (3)
C17—C18	1.388 (3)	C47—C48	1.410 (4)
C18—H18	0.9500	C47—O8	1.373 (3)
C19—C20	1.433 (3)	C48—C49	1.391 (3)
C19—N4	1.382 (3)	C48—O7	1.349 (3)
C20—H20	0.9500	C49—H49	0.9500
C20—C21	1.351 (3)	C49—C50	1.387 (4)
C21—H21	0.9500	C50—H50	0.9500
C21—C22	1.438 (3)	C51—H51A	0.9800
C22—C23	1.399 (3)	C51—H51B	0.9800
C22—N4	1.378 (3)	C51—H51C	0.9800
C23—C24	1.506 (3)	C51—O8	1.430 (3)
C23—C30	1.403 (3)	O7—H7A	0.8400
C24—C25	1.386 (3)	C52—S0AA	1.812 (3)
C24—C29	1.394 (3)	C52—F1_1	1.342 (5)
C25—H25	0.9500	C52—F2_1	1.431 (5)
C25—C26	1.397 (3)	C52—F3_1	1.259 (4)
C26—H26	0.9500	C52—F1A_2	1.315 (6)
C26—C27	1.379 (3)	C52—F2A_2	1.293 (5)
C27—C28	1.377 (3)	C52—F3A_2	1.403 (6)
C27—Cl3	1.751 (2)	O3—S0AA	1.4402 (18)
C28—H28	0.9500	O4—S0AA	1.4380 (19)
C28—C29	1.389 (3)	O5—S0AA	1.437 (2)
C29—H29	0.9500	O6—H6A	0.8699
C30—C31	1.436 (3)	O6—H6B	0.8702
C30—N3	1.379 (3)	O10—H10A	0.8696
C31—H31	0.9500	O10—H10B	0.8701
C31—C32	1.354 (3)	O10B—H10C	0.8703
C32—H32	0.9500	O10B—H10D	0.8697
C32—C33	1.438 (3)		
			
C8—C1—C2	118.1 (2)	C35—C36—H36	119.7
C43—C1—C2	116.9 (2)	C35—C36—C37	120.6 (2)
C43—C1—C8	125.0 (2)	C37—C36—H36	119.7
C3—C2—C1	119.6 (2)	C36—C37—H37	120.4
C7—C2—C1	121.7 (2)	C38—C37—C36	119.2 (2)
C7—C2—C3	118.7 (2)	C38—C37—H37	120.4
C2—C3—H3	119.6	C4EA—C38—Cl2	118.87 (18)
C4—C3—C2	120.8 (2)	C37—C38—C4EA	121.5 (2)
C4—C3—H3	119.6	C37—C38—Cl2	119.65 (18)
C3—C4—H4	120.4	C4EA—C39—C35	121.1 (2)
C5—C4—C3	119.2 (2)	C4EA—C39—H39	119.5
C5—C4—H4	120.4	C35—C39—H39	119.5
C38—C4EA—H4EA	120.6	C34—C40—C41	124.7 (2)
C38—C4EA—C39	118.8 (2)	N2—C40—C34	126.0 (2)
C39—C4EA—H4EA	120.6	N2—C40—C41	109.33 (19)
C4—C5—C6	121.7 (2)	C40—C41—H41	126.2
C4—C5—Cl1	118.26 (19)	C42—C41—C40	107.6 (2)
C6—C5—Cl1	120.00 (19)	C42—C41—H41	126.2
C5—C6—H6	120.6	C41—C42—H42	126.3
C5—C6—C7	118.8 (2)	C41—C42—C43	107.3 (2)
C7—C6—H6	120.6	C43—C42—H42	126.3
C2—C7—H7	119.6	C1—C43—C42	125.5 (2)
C6—C7—C2	120.8 (2)	N2—C43—C1	125.4 (2)
C6—C7—H7	119.6	N2—C43—C42	109.03 (19)
C1—C8—C9	125.0 (2)	C8—N1—C11	106.51 (17)
N1—C8—C1	125.8 (2)	C8—N1—Fe1	126.67 (15)
N1—C8—C9	109.22 (19)	C11—N1—Fe1	126.81 (14)
C8—C9—H9	126.4	C40—N2—C43	106.70 (18)
C10—C9—C8	107.2 (2)	C40—N2—Fe1	126.14 (15)
C10—C9—H9	126.4	C43—N2—Fe1	127.04 (15)
C9—C10—H10	126.1	C30—N3—Fe1	126.49 (15)
C9—C10—C11	107.8 (2)	C33—N3—C30	106.68 (18)
C11—C10—H10	126.1	C33—N3—Fe1	126.64 (14)
C12—C11—C10	125.1 (2)	C19—N4—Fe1	126.93 (14)
N1—C11—C10	109.22 (19)	C22—N4—C19	106.61 (18)
N1—C11—C12	125.69 (19)	C22—N4—Fe1	126.34 (15)
C11—C12—C13	117.66 (19)	H1A—O1—H1B	107.9
C11—C12—C19	125.0 (2)	Fe1—O1—H1A	111.0
C19—C12—C13	117.27 (19)	Fe1—O1—H1B	110.7
C14—C13—C12	122.3 (2)	H2A—O2—H2B	110.5
C14—C13—C18	118.5 (2)	Fe1—O2—H2A	110.5
C18—C13—C12	119.2 (2)	Fe1—O2—H2B	95.4
C13—C14—H14	119.7	N1—Fe1—N2	89.77 (7)
C13—C14—C15	120.7 (2)	N1—Fe1—N3	177.28 (7)
C15—C14—H14	119.7	N1—Fe1—N4	89.88 (7)
C14—C15—H15	120.4	N1—Fe1—O1	92.61 (7)
C16—C15—C14	119.3 (2)	N1—Fe1—O2	88.79 (7)
C16—C15—H15	120.4	N2—Fe1—N3	90.03 (7)
C15—C16—Cl4	119.78 (19)	N2—Fe1—N4	176.22 (8)
C17—C16—C15	121.4 (2)	N2—Fe1—O1	92.13 (7)
C17—C16—Cl4	118.86 (19)	N2—Fe1—O2	87.49 (7)
C16—C17—H17	120.5	N3—Fe1—O1	90.11 (7)
C16—C17—C18	118.9 (2)	N3—Fe1—O2	88.49 (7)
C18—C17—H17	120.5	N4—Fe1—N3	90.14 (7)
C13—C18—H18	119.4	N4—Fe1—O1	91.64 (7)
C17—C18—C13	121.3 (2)	N4—Fe1—O2	88.74 (7)
C17—C18—H18	119.4	O1—Fe1—O2	178.54 (7)
C12—C19—C20	125.2 (2)	C45—C44—H44	117.0
N4—C19—C12	125.5 (2)	O9—C44—H44	117.0
N4—C19—C20	109.34 (19)	O9—C44—C45	125.9 (3)
C19—C20—H20	126.3	C46—C45—C44	120.6 (2)
C21—C20—C19	107.4 (2)	C50—C45—C44	119.2 (2)
C21—C20—H20	126.3	C50—C45—C46	120.2 (2)
C20—C21—H21	126.2	C45—C46—H46	120.5
C20—C21—C22	107.5 (2)	C47—C46—C45	119.0 (2)
C22—C21—H21	126.2	C47—C46—H46	120.5
C23—C22—C21	125.1 (2)	C46—C47—C48	120.7 (2)
N4—C22—C21	109.11 (19)	O8—C47—C46	125.5 (2)
N4—C22—C23	125.8 (2)	O8—C47—C48	113.8 (2)
C22—C23—C24	117.35 (19)	C49—C48—C47	119.9 (2)
C22—C23—C30	125.5 (2)	O7—C48—C47	116.2 (2)
C30—C23—C24	117.06 (19)	O7—C48—C49	123.9 (2)
C25—C24—C23	122.1 (2)	C48—C49—H49	120.4
C25—C24—C29	119.0 (2)	C50—C49—C48	119.3 (2)
C29—C24—C23	118.90 (19)	C50—C49—H49	120.4
C24—C25—H25	119.5	C45—C50—C49	121.0 (2)
C24—C25—C26	121.0 (2)	C45—C50—H50	119.5
C26—C25—H25	119.5	C49—C50—H50	119.5
C25—C26—H26	120.8	H51A—C51—H51B	109.5
C27—C26—C25	118.5 (2)	H51A—C51—H51C	109.5
C27—C26—H26	120.8	H51B—C51—H51C	109.5
C26—C27—Cl3	119.78 (18)	O8—C51—H51A	109.5
C28—C27—C26	121.9 (2)	O8—C51—H51B	109.5
C28—C27—Cl3	118.34 (18)	O8—C51—H51C	109.5
C27—C28—H28	120.5	C48—O7—H7A	109.5
C27—C28—C29	119.0 (2)	C47—O8—C51	117.1 (2)
C29—C28—H28	120.5	F1_1—C52—S0AA	107.0 (3)
C24—C29—H29	119.7	F1_1—C52—F2_1	102.0 (3)
C28—C29—C24	120.7 (2)	F2_1—C52—S0AA	106.9 (2)
C28—C29—H29	119.7	F3_1—C52—S0AA	120.7 (3)
C23—C30—C31	125.3 (2)	F3_1—C52—F1_1	112.0 (3)
N3—C30—C23	125.4 (2)	F3_1—C52—F2_1	106.5 (3)
N3—C30—C31	109.31 (19)	F1A_2—C52—S0AA	115.5 (3)
C30—C31—H31	126.3	F1A_2—C52—F3A_2	105.1 (4)
C32—C31—C30	107.4 (2)	F2A_2—C52—S0AA	113.6 (3)
C32—C31—H31	126.3	F2A_2—C52—F1A_2	111.6 (4)
C31—C32—H32	126.4	F2A_2—C52—F3A_2	105.3 (3)
C31—C32—C33	107.3 (2)	F3A_2—C52—S0AA	104.6 (3)
C33—C32—H32	126.4	O3—S0AA—C52	104.66 (12)
C34—C33—C32	125.4 (2)	O4—S0AA—C52	104.07 (14)
N3—C33—C32	109.33 (19)	O4—S0AA—O3	115.50 (12)
N3—C33—C34	125.22 (19)	O5—S0AA—C52	102.84 (14)
C33—C34—C35	119.17 (19)	O5—S0AA—O3	114.19 (12)
C33—C34—C40	125.4 (2)	O5—S0AA—O4	113.65 (12)
C40—C34—C35	115.39 (19)	H6A—O6—H6B	109.5
C36—C35—C34	121.8 (2)	H10A—O10—H10B	109.4
C36—C35—C39	118.8 (2)	H10C—O10B—H10D	109.5
C39—C35—C34	119.2 (2)		

### Hydrogen-bond geometry (Å, º)

Cg2, Cg3, Cg4, Cg10 and Cg13 are the centroids of the N2/C40–C43, N3/C30–C33, N4/C19–C22, C2–C7 and C45–C50 rings, respectively.

**Table:**

*D*—H···*A*	*D*—H	H···*A*	*D*···*A*	*D*—H···*A*
O1—H1*A*···O10*B*^i^	0.89	1.74	2.626 (8)	171
O1—H1*B*···O9	0.89	1.96	2.747 (2)	146
O2—H2*A*···O3^ii^	0.88	1.83	2.705 (2)	171
O6—H6*A*···O7^iii^	0.87	2.45	3.064 (3)	128
O6—H6*A*···O8^iii^	0.87	2.11	2.946 (3)	162
O6—H6*B*···O4^iv^	0.87	1.93	2.790 (3)	168
O7—H7*A*···O6^v^	0.84	1.77	2.596 (3)	167
O10—H10*A*···O7^vi^	0.87	2.07	2.92 (3)	165
O10*B*—H10*C*···O5^vii^	0.87	1.92	2.786 (7)	177
O10*B*—H10*D*···O7^vi^	0.87	2.02	2.876 (5)	166
C10—H10···Cl3^viii^	0.95	2.76	3.659 (2)	159
C14—H14···O5^ix^	0.95	2.38	3.297 (3)	162
C31—H31···Cl2^i^	0.95	2.82	3.739 (2)	163
C4*EA*—H4*EA*···*Cg*4^x^	0.95	2.66	3.5054	149
C17—H17···*Cg*2^ii^	0.95	2.76	3.5715	144
C20—H20···*Cg*13^ii^	0.95	2.82	3.5054	130
C28—H28···*Cg*3^xi^	0.95	2.79	3.6574	152
C37—H37···*Cg*10^xii^	0.95	2.76	3.6107	149

Symmetry codes: (i) −*x*+1, −*y*+1, −*z*+1; (ii) *x*+1, *y*, *z*; (iii) −*x*, −*y*+1, −*z*+1; (iv) −*x*, *y*−1/2, −*z*+1/2; (v) *x*, *y*+1, *z*; (vi) *x*, *y*−1, *z*; (vii) *x*, −*y*+1/2, *z*+1/2; (viii) *x*, −*y*+3/2, *z*−1/2; (ix) −*x*+1, *y*+1/2, −*z*+1/2; (x) *x*−1, *y*, *z*; (xi) −*x*+2, −*y*+1, −*z*+1; (xii) −*x*+1, *y*−1/2, −*z*+1/2.
